# Middle-latency auditory responses in neurological diseases

**DOI:** 10.1016/S1808-8694(15)30107-5

**Published:** 2015-10-19

**Authors:** Paulo Roberto Pialarissi, Francisco S. Almeida, Lucrécia C.B.M. Camanducaia, Jose Jarjura Jorge

**Affiliations:** a PhD - University of São Paulo. Full Professor - PUC-SP, Professor of the Speech and Hearing Department - PUC-SP.; b PhD in Otorhinolaryngology - University of São Paulo. Coordinator of the Otorhinolaryngology Service - Hospital Odontomed de Itajubá.; c MD. Neurosurgeon - Hospital Escola da Faculdade de Medicina de Itajubá, Minas Gerais.; d Full Professor - FCMB - PUC-SP. Head of the ENT Discipline - PUC-SP. Pontifícia Universidade Católica de São Paulo.

**Keywords:** central auditory evaluation, neurological diseases, auditory eletrophysiology, middle latency auditory evoked potentials

## Abstract

The presence of middle-latency evoked auditory potentials allows for integrity evaluation of both peripheral and the central auditory systems, and also, that of the nucleus and auditory pathways of sub-cortical region. They have also been used to study alterations of these structures in different neurological diseases.

**Aims:**

the aim of this study is to verify the latency of the middle-latency evoked auditory potentials and detect the presence of any deflections in subjects with neurological diseases.

**Materials and Methods:**

In a clinical and prospective trial, 20 patients having central neurological diseases of various etiologies were evaluated and, positive and negative deflections produced by the middle-latency evoked auditory potentials in these patients were analyzed.

**Results:**

Data was statistically analyzed and showed significant modifications in middle-latency evoked auditory potentials.

**Conclusion:**

The authors concluded that patients with neurological disorders have either wave suppression or enlarged latency periods in relation to normal subjects.

## INTRODUCTION

Obtaining middle latency auditory evoked potentials is highly important in an attempt to improve the objectiveness of assessing patients with hearing loss, both in regards of determining their thresholds, as well as in locating the lesion. Today, they have also been used to study the alterations that happen in different neurological disorders.

These potentials represent a series of deflections that happen between 10 and 80 milliseconds (ms) after the auditory stimulus, and are located beyond brainstem evoked potentials, preceding later responses, which are related to cortical and cognitive functions.

These responses were firstly recorded at the “Massachusetts Institute of Technology” by Geisler et al.^1^, in 1958, at the time they used a computerized device to measure the responses. These authors inferred at the time that the deflections observed in their work represented afferent auditory activities associated with the anterior regions of the cerebral cortex.

Nonetheless, in 1963, Bickford et al., stated that these responses were purely generated by muscle potentials^2^. With that, they stopped studying these potentials as useful to assess afferent auditory pathways.

Starting in 1967, studies^3^ were carried out showing the clinical applicability of these responses. This was reinforced after researchers started using surface electrodes in patients who underwent neurosurgery^4^.

In 1974, many waves were described, such as N18 (Na), P30 (Pa) and P50 (Pb), trying to separate these responses from the pure myogenic responses, such as the ones that occur by contraction of the post-auricular and temporal muscles^5^.

Later studies^6^ showed the presence of these deflections even when there was muscle paralysis obtained through the administration of succinylcholine. These data were reinforced by using an anesthetic agent (Fentanyl) associated with “pancuroniom”, which caused muscle anesthesia^7^.

From then on, many authors carried out studies on the sites that generate deflections with latencies between 10 and 80ms. It was so demonstrated that wave Pa suffers a clear reduction in patients with temporal lobe lesion, when compared to the normal hemisphere^8^, and through animal studies, they were able to differentiate the primary sensorial portions from those originating in the extra-lemniscuses pathways of the auditory system^9^ (the reticular substance, for example), suggesting the importance of the thalamic-cortical pathways.

The many deflections are described in details in relation to their sites of generation, using surface electrodes in patients undergoing neurosurgeries^10^.

Despite these evidences, more systematized studies about these responses were made to assess afferent auditory pathways, studies that only started to gain some ground at the late 80’s.

In 1990, these middle latency potentials are characterized between 20 and 70ms, both with monoauricular and binauricular stimulation, with the following distribution: Pa, the best established middle latency response, at 29ms; Pb at 53ms and Tp at 41ms. Intracranial pharmacological and topographical evaluation, and those by induced lesions indicate that these three positive deflections are of neural origin. It is still unclear if Pa and/or Pb are produced in the Heschl gyrus, in the primary cortex; while Tp is probably generated by the auditory cortex, on the lateral surface of the temporal lobe^11^.

In studies carried out with lab animals, it was shown that responses obtained at the ventral and caudal-medial subdivisions of the medial geniculate body are functionally different. The inhibitory effect on recordings obtained from the ventral subdivision is similar to the one seen in middle latency components recorded on the temporal cortex, while the recording obtained from the caudal-medial subdivision is similar to the ones in the middle line. Moreover, these imply that binauricular patterns seen in the primary and non-primary auditory cortex may be processed and coded in the thalamus^12^.

Other studies showed that the generators of the components obtained at 30, 50, 60 and 75ms are distributed medio-lateraly along the Heschl gyrus^13^.

It is believed that the generation of middle latency auditory potentials may reflect an interface of primary and non-primary areas of the thalamic-cortical auditory pathways. Primary and non-primary components may be differentiated in numerous ways: by lesion, by stimuli variations and topographically. Non-primary components develop early on and are likely to be dependent on sleep status, while primary components develop later on and are detectable during sleep^14^.

Thus, it is characterized that middle latency auditory responses, are a sequence of evoked potentials, which occur within a given time span, with latencies of 10 to 60ms. These potentials would have a relatively long development time, which extends through a person’s first decade of life. We then describe the characteristics of development alterations for each one of these deflections, not only related to their formats, but also to the potential reproducibility, alert status dependence and stimuli rates. Studies both in humans and animals indicate that these complex changes may be the result of multiple generator systems that have different development stages^15^.

Obtaining middle latency auditory potentials start having major clinical applications in different situations, such as in the electrophysiological determination of auditory thresholds in low frequencies, in assessing cochlear implant functioning, assessing the functioning of the auditory pathways, in locating lesions in the auditory pathways and in intra-operative applications^16^.

These evoked potentials may be used to assess information processing. When we analyze somatosensory and auditory middle latency auditory potentials concurrently^17^, most specifically P50, in healthy males, it is suggested a support for the information processing deficiency theory in individuals with schizophrenia (defect in P50).

In an attempt to explore other aspects of information processing, there are some researchers studying the habituation of auditory evoked responses (P50) with the use of repetitive stimuli^18^. It is observed that the P50 response amplitude to the second of two homologous stimuli was significantly less reduced in patients with migraine when compared to healthy volunteers.

Still in 2001, based on middle latency evoked auditory responses seen in patients with Obstructive Sleep Apnea Syndrome, before and after treatment, it is suggested that the ascending reticular activity may be involved in these patients^19^. After treating apnea, there was a significant improvement in nocturnal hypoxia and there also was an increase in P1 peak of middle latency, and also these potentials had a better distribution in their electrical field in the scalp.

In this same year, a study was carried out in which they obtained brain stem auditory responses and also middle latency auditory responses in three groups: one from patients with tinnitus, another one from normal individuals and, the third, from elderly patients^20^. In the groups of tinnitus patients and elderly patients, as far as brain stem potentials are concerned, we observed alterations in relation to wave VII, and in terms of medium latency potentials, many wide deflections were observed occurring in some of this group’s components, but not in all of them. There still was a widening of these middle deflections without the corresponding widening in brain stem potentials. In our opinion, this suggests there could be a selective alteration in the middle latency deflections generators in patients with tinnitus and different age effects on cochlear physiology.

As they researched using two comparative methods, obtaining middle latency evoked auditory responses by electroencephalographic recordings or by means of magnetoencephalographics, multiple supratemporal bypasses were suggested for the different deflections observed. Therefore, Pa (28ms) is correlated to the medial portion of the Heschl gyrus; Nb (40ms)/ Pb1(52ms) to the lateral face of the supratemporal gyrus; and Pb2 (74ms) to the antero-lateral portion of the Heschl gyrus^21^. They argued that these findings are in accordance with prior invasive intracerebral recordings and with animal studies, which reported the secondary areas involved in the generation of middle latency evoked auditory components.

Later on, carrying out a simultaneous capture of intracranial auditory evoked potentials directly from the auditory cortex and the medial geniculate body^22^, from a patient, the same researchers noticed an initial negative response generated at the level of the medial geniculate body, with latency of about 13.5ms, and two positive peaks (P21 and P29), with higher amplitudes for low tones and suggested the existence of a possible tonotopic organization of this nucleus. They also observed that peaks originating from the thalamus activity were strongly interlaced with the cortical activities recorded in the Heschl gyrus before 30ms (N13 precedes the first cortical component in 3.5ms; while P21 and P29 precede and delays, respectively, in relation to the two following cortical responses that have reverse polarity in an interval of 1.5 - 2ms. This study provides new functional data on the medial geniculate body activity and suggests a more complex role for the thalamus insofar as sound perception is concerned.

In our country, in 2003, a study considered critical the need to understand the maturation of the auditory system when one wants to check the integrity of central auditory pathways. When we studied middle latency potentials in 155 normal individuals from 07 to 16 years of age, we noticed that it was possible to observe a Pa wave in all individuals tested; however, it was not possible to check central nervous system maturity through its latency and amplitude^23^.

When middle latency potentials were analyzed in patients with myotonic dystrophy and central nervous system involvement, one notices that Na and Pa amplitudes are significantly higher when compared to the ones recorded from normal individuals ^24^.

It has been shown the importance of recording different accessible potentials in order to monitor the functional status of the central nervous system when assessing cortical dysfunctions resulting from hemorrhagic, ischemic or hypoxic strokes^25^.

Brainstem evoked auditory potentials are altered in pregnant diabetic women when compared to normal pregnant women. In the former, latencies were observed in deflections I to V and interpeak latencies I-III and I-V were significantly higher, while wave V amplitude was reduced. Nonetheless, we did not find any significant alteration in the latencies of medium potentials^26^.

These potentials represent an important tool to assess cerebral function, not only from the auditory standpoint, but also in patients with neurological disorders, as it has been shown in many studies with comatose patients or patients who suffered a head injury. Prognostic values of the somatosensorial and auditory cortex evoked responses are evaluated in comatose patients.^27^

In a population of 27 patients with schizophrenia, when compared to the normal population, alterations were detected in the form of middle latency deflections, and also increased latencies. It is suggested that the morphological abnormalities of middle latencies in schizophrenia are significant and must be examined in this population^28^.

Investigating the P50 deflection in patients with post-traumatic disorders secondary to urban violence^29^, and comparing it to normal individuals and schizophrenic patients, we can notice that these potentials bear the same parameters as those observed for patients with schizophrenia and are lower than the ones observed in normal subjects. Thus, it is suggested that the P50 paradigm may also become an objective parameter used to assess the new treatment modalities for post-traumatic disorders.

In recent years, many scientific investigations have been carried out, showing that these potentials are related to the auditory nuclei and pathways located on the subcortical and primary cortical regions, especially in the thalamic-cortical tract.

## GOALS

Our study of middle latency evoked auditory potentials in a sample of individuals with neurological disorders, with central involvement (brainstem and subcortical regions), had the following goals:

1. Assess the presence of the many deflections originated from medium latency auditory potentials.
2. Compare the values of their latencies, as well as that from the Na-Pa interval, with the values found in a normal, standardized, previously studied population.

## MATERIALS AND METHODS

Our series counted on 20 individuals, from both genders, without a pre-determined age, who had neurological disorders, of different etiologies of a central level, who were neurologically, otologically and audiologically assessed. These evaluations were not invasive and, usually, do not cause any harm to the individual.

All the individuals participating in this study were duly informed about the procedures to which they would be submitted, as well as the study’s goal, and they signed an informed consent.

This study was quantitative and the sampling was non-probabilistic. The study subjects were chosen by convenience and the data collected were assessed taking into account inter-individual relations. We used a cohorte approach, with contemporary evaluation.

We applied descriptive and inference statistical studies. In them, data were analyzed having in mind the “T student”, bi-caudal, test, with equal sample variance, considering a significance level (p) below or equal to 0.05.

The study that involved human beings followed strict ethical rules that were established and was submitted to the Ethics Committee of the institution under protocol # 201/04.

The selection process counted on anamnesis, neurological assessment, clinical otological inspection, acoustic immittance measures, stapedial reflexes study, conventional tonal audiometry and logoaudiometry. When necessary, it was complemented with other tests, such as electroencephalography and image evaluations (CT scan; MRI; angio-resonance and/or “echodoppler” of carotid and vertebral arteries.

All individuals were submitted to the capture of Brainstem Evoked Auditory Potentials and Medium Latency Evoked Auditory Potentials.

Brainstem Potentials and Middle Latency Potentials capture were carried out with the equipment and the individuals inside a shielded acoustic booth, in order to avoid electrical interference of any nature (for example static electricity).

With that we would only have left the interferences belonging to the individuals that were tested or the examiner. We also tried to avoid them by means of additional care. The electrodes, as well as the ear phones were positioned always by the same examiner, after proper individual education. Booth environmental conditions (temperature, lighting, silence and positioning) were the most adequate ones to keep the subject calm and relaxed, without letting him/her sleep. The electrodes were attached after careful skin cleaning, with proper electrolytic paste, so as to better capture the potentials of interest. The test only started when the baseline electroencephalogram was stable and without interferences.

The equipment used was the CE - EP 25 operational system. The CE brand indicates that “Interacoustics” fulfills the demands required in attachment VI of medical guideline 93/42/EEC and was approved by TÜV, with identification # 0123.

The exam protocol used in our research for the capture of middle latency potentials is the following:

- Stimulus: “Click”, lasting for 100ms, with alternate polarity, without masking, at the frequency of 7.0 stimuli per second, in a total of 1000 stimuli.
- Stimulus intensity: 70 dB SPL, introduced through TDH 39 ear phones.
- Electrodes: Two “Live” electrodes, placed on the upper region of the scalp, at half the distance between the cranial vault vertex and the mastoid region, in each side; two “reference” electrodes, were placed in the mastoid region, on each side, and a “ground” electrode was placed on the glabella.
- Impedance: electrodes impedance was around 2W, and a maximum of 3W is accepted.
- Recording time span: from 0 to 80ms.
- Recording time span: from 0 to 80ms.Filters: 10 Hz high pass and 1200 Hz low pass.
- Recording time span: from 0 to 80ms.Reproducibility index: 95%.
- Recording time span: from 0 to 80ms.We used mono-auricular stimulation.
- Recording time span: from 0 to 80ms.Stimuli and response capture sequence:
  1. Stimulus: Right Ear Capture: Right Hemisphere (RERH)
  2. Stimulus: Left Ear Capture: Right Hemisphere (LERH)
  3. Stimulus: Right Ear Capture: Left Hemisphere (RELH)
  4. Stimulus: Left Ear Capture: Left Hemisphere (LELH)

## RESULTS

The patients evaluated, in a total of 20, in relation to their respective neurological disorders, are detailed on Table 1.

**Table 1:** List of patients who participated in this study and their respective neurological diagnosis.

Patient	Initials	Neurological Disorder
01	BPS	Cerebral metastasis in the left hemisphere (03 nodules) - renal primary tumor
02	MZG	Cavernous sinus tumor
03	GMB	Left side ischemic stroke
04	AKH	Left side ischemic stroke
05	EFVB	Aneurism of the left carotid artery (posterior communicating)
06	AMRB	Multiple sclerosis
07	MIC	Cerebral atrophy
08	NMMA	Hydrocephaly and infection of the pellucid septum - with lesions to the cranial nerves I, II, III, IV.
09	MLSN	Parkinson’s disease
10	SDS	Hydrocephaly
11	JLO	Congenital anomaly - arachnoid cyst
12	THRM	Depression - psychiatric patient
13	EBR	Subdural hematoma in the parietal region
14	NFS	Right side ischemic stroke
15	BMP	Subarachnoid hematoma
16	BBC	Temporal-parietal expansive lesion and paroxysmal activity of diffuse projection
17	CCS	Psychosis and chronic chemical intoxication
18	AJS	Tumor in the pontine-cerebellar angle
19	AMRCT	Demyelinating disease
20	NAP	Demyelinating disease

After submitting these patients to the predicted exams, the records obtained were analyzed and plotted on the following tables.

Table 2 shows the minimum and maximum values in the periods of deflection latency Po, Na, Pa, Nb and Pb, as well as from interval Na-Pa (in ms), with their mean values and standard deviations; such deflections were generated with acoustic stimuli in each ear, respectively and with their records being obtained specifically for each cerebral hemisphere.

**Table 2:** Nb and Pb, as well as from interval Na - Pa (in ms), with their mean value and standard deviation using acoustic stimuli in each ear respectively, and obtaining records specifically for each cerebral hemisphere.

Stimulus X Capture	Statistical analysis	Po	Na	Pa	Nb	Pb	Na-Pa
	Valid #	20	20	20	20	20	20
	Minimum	9,33	11,33	21,67	32,33	43,33	5,33
RERH	Maximum	18,67	25,33	45,33	67,00	74,67	27,67
	Mean value	12,23	17,57	32,98	48,98	59,40	15,42
	St. Deviation	2,32	3,61	5,70	9,71	9,43	5,69
	Valid #	17	17	17	17	17	17
	Minimum	9,33	13,00	22,33	28,33	36,00	5,33
RELH	Maximum	17,33	26,33	50,67	63,33	77,00	31,33
	Mean value	12,45	19,37	33,84	47,27	58,53	14,47
	St. Deviation	2,08	3,27	7,17	10,29	14,43	7,84
	Valid #	16	16	16	16	16	16
	Minimum	10,00	11,67	22,67	27,33	39,00	9,00
LERH	Maximum	21,00	32,33	43,00	63,00	79,00	27,00
	Mean value	13,52	19,02	32,90	47,52	61,65	13,87
	St. Deviation	3,18	5,12	5,37	11,74	13,44	5,07
	Valid #	19	19	19	19	19	19
	Minimum	8,67	15,33	23,67	33,67	40,33	4,00
LELH	Maximum	16,33	35,00	56,67	67,33	77,33	35,33
	Mean value	12,56	19,63	34,56	49,21	55,82	14,93
	St. Deviation	1,88	4,22	7,05	12,39	11,48	6,77

Table 3 shows the data aforementioned, grouped in totals.

**Table 3:** Minimum and maximum values of the latency periods of latencies from deflections Po, Na, Pa, Nb and Pb, as well as in interval Na - Pa (in ms), with mean value and standard deviation, grouped, in relation to the stimulated ear and the cerebral hemisphere, place of its capture.

Stimulus X Capture	Statistical analysis	Po	Na	Pa	Nb	Pb	Na-Pa
	Valid #	72	72	72	72	72	72
	Minimum	8,67	11,33	21,67	27,33	36,00	4,00
All	Maximum	21,00	35,00	56,67	67,33	79,00	35,33
	Mean value	12,66	18,86	33,58	48,31	58,75	14,72
	St. Deviation	2,38	4,08	6,28	10,86	12,11	6,32

Table 4 analyzes the significance of the values obtained, crossing the different records obtained in each cerebral hemisphere with the stimulus being given separately in each ear.

**Table 4:** Significance analysis of the values obtained in relation to the latency periods of deflections Po, Na, Pa, Nb, Pb and the interval Na-Pa, crossing the different recordings obtained in each cerebral hemisphere and with the stimulus being given to each ear separately.

Stimulus X Capture	Po	Na	Pa	Nb	Pb	Na-Pa
RERH x RELH	0,77	0,12	0,69	0,61	0,83	0,67
LERH x LELH	0,28	0,70	0,44	0,68	0,18	0,61
RERH x LERH	0,17	0,32	0,96	0,68	0,56	0,40
RELH x LELH	0,87	0,84	0,76	0,62	0,54	0,85
RERH x LELH	0,63	0,11	0,45	0,95	0,29	0,81
RELH x LERH	0,26	0,81	0,67	0,95	0,53	0,80

The statistical comparison between the results obtained from the group with neurological disorders and the normal group, previously standardized can be seen on Table 5.

**Table 5:** Comparison of the mean values of latency periods from deflections Po, Na, Pa, Nb and Pb, as well as from interval Na - Pa (in ms), between the normal group, previously standardized and the group of patients with neurological disorders.

	Po	Na	Pa	Nb	Pb	Na-Pa
Normal Standard	12,09	17,91	29,41	41,43	51,44	11,52
Neurological disorders	12,66	18,86	33,58	48,31	58,75	14,72
p	0,041	0,024	0,000	0,000	0,000	0.009

## DISCUSSION

The capture of middle latency evoked auditory potentials, described approximately five decades ago, was the topic of much controversy during a long time in regards of its origin (neural or purely muscular)^1^, ^2^, ^3^, ^4^, ^5^, ^6^, ^7^.

Many authors have performed research with normal individuals using surface electrodes or placed inside the cranial cavity during a surgical procedure in human beings,^10^, ^22^ or in animals,^9^ in order to determine the standards in relation to the medium latency auditory evoked potential records. Through electrophysiological, topographical, pharmacological methods and lesions, it has been shown that the region of origin of these potentials is located in the Heschel’s gyrus^11^, ^13^, in the primary cortex. Studies both in humans and in animals indicate that these complex changes may be the result of multiple generating systems that show different stages of development^12^, ^15^.

Some authors, by means of electroencephalographic records and magnetoencephalographic data suggest multiple supratemporal sources for the many deflections observed. They then correlate Pa (28ms) to the medial portion of the Heschl’s gyrus; Nb (40ms)/ Pb1(52ms) to the lateral face of the supratemporal region; and Pb2 (74ms) to the antero-lateral portion of the Heschl’s gyrus^21^.

It is then believed that these responses originate from the auditory and non-auditory primary cortical area, especially from the thalamic-cortical bundles^12^, ^15^, ^22^. This region is fundamental in the transmission of auditory impulses to the cerebral cortex, where the sound message decoding process must happen. This decoding is essential for vocal discrimination and for human communication. This area also involves other cerebral systems, affecting other sensory and motor functions. This leads us to think that the recordings of these responses may become important as a means to assess and diagnose different neurological diseases. These statements serve as a basis for our study.

The clinical applicability of these potentials are found in many situations, such as, in the electrophysiological determination of auditory thresholds in the range of low frequencies^25^, in assessing the functioning of the cochlear implant, in assessing auditory pathways functioning, in mapping neurological lesions and in intraoperative monitoring16. These evoked potentials may be used to assess information processing^17^, ^18^ or in patients with tinnitus^20^, in relation to the auditory system.

In this study we noticed that these potentials make up an important tool for cerebral function evaluation, not only from the auditory standpoint, but also in patients with neurological disorders, as it has been shown in many studies with comatose patients or those who suffered head injuries, including prognosis,^17^, ^19^, ^24^, ^26^, ^27^, ^28^, ^29^. These authors studied different neurological disorders showing the existing alterations in middle latency evoked auditory potential parameters.

It is paramount to understand auditory system maturation when one wishes to check the integrity of the central auditory pathways^23^.

In this study we grouped neurological disorders of different etiologies and we compared the results attained from the normal group previously assessed in our service^30^.

Through statistical comparison among the results found (deflection latencies Po, Na, Pa, Nb, Pb and the Na-Pa interval) in the groups of neurological disorders and the normal group, previously standardized, it was established that the mean values are significantly higher in the first group. The most significant statistical differences were mainly related to the Pa, Nb and Pb deflections.

By crossing the values obtained from the recordings of each cerebral hemisphere, with auditory stimulation in each ear, respectively, we did not find statistically significant differences as to the site of response capture, and this allows the exam to be carried out without the concern of where the electrodes will be placed.

We can see that the recording of these potentials provide a reliable basis for the assessment of dysfunctions or even lesions in the central nervous system. In this study we did not sequentially follow up the patients, therefore there are no grounds in order to assess prognosis.

During the exams, we concurrently captured the middle latency auditory evoked responses and also the short latency auditory evoked responses. At this time, we did no analyze these results comparatively. Notwithstanding, this facility makes this exam an extraordinary tool of great importance in daily clinical practice.

There was no concern in specifically analyzing the characteristics of each disorder separately. The goal of the study was to analyze the results obtained and the alterations seen in the whole set of diseases present in the patients examined. This is important, considering the different diseases that may cause diffuse alterations in the structure and functioning of the cerebral tissue.

The investigation of these middle latency potentials presents a great study possibility from the auditory and neurological standpoint. It allows us to perform an electrophysiological assessment of the subjects hearing level, and also provides for the identification of the possible lesion side, in the case of neurological disorders.

## CONCLUSIONS

In our sample of patients with neurological disorders, in relation to the deflections generated by the middle latency auditory potentials, we concluded that:

1. Almost all the recordings were captures, there was suppression in only a few of them.
2. The maximum and middle values of their latency periods, as well as the Na-Pa intervals, were significantly increased when compared to the ones obtained from a normal standardized population in our settings.

## References

[1] Geisler CD, Frishkopf LS, Rosenblith WA (1958). Extracranial responses to acoustic clicks in man. Science.

[2] Bickford RG, Galbraith RF, Jacobson JL (1963). The nature of averaged evoked potentials recorded from the human scalp. Electroenceph Clin Neurophysiol.

[3] Goldstein R, Rodman LB (1967). Early components fo averaged evoked responses to rapidly repeats auditory stimuli.. J. Speech Hear Res.

[4] Celesia GG, Broughton RJ, Rasmussen TH, Branch C (1968). Auditory evoked response from the exposed human cortex. Electroenceph Clin Neurophisiol.

[5] Picton TW, Hillyard SA, Krausz HI, Galambos R (1974). Human auditory evoked potentials. Evaluation of components. Electroenceph Clin Neurophysiol.

[6] Harker E, Hosick E, Voots RJ, Mendel M. (1977). Influence of succinilcholine on middle component auditory evoked potentials. Arch Otolaryngol.

[7] Kileny PR, Berry DA, Menches G, Gerber S. (1983). The Multiply Handicapped Hearing Impaired Child.

[8] Kraus N, Ozdamar O, Hier K, Stein L (1982). Auditory middle latency responses im patients with cortical lesions. Electroenceph Clin Neurophysiol.

[9] Mcgee T, Ozdamar O, Kraus N (1983). Auditory middle latenccy responses in the guinea pig. Am J Otolaryngol.

[10] Lee YS, Lueders H, Dinner DS, Lesser RP, Hahn, J, Klen G. (1984). Recording of auditory evoked potentials in man using chroni subdural electrodes. Brain Res.

[11] Cacace AT, Satya-Murti S, Wolpaw JR (1990). Human middle-latency auditory evoked potentials: vertex and temporal components. Electroencephalography and clinical Neurophysiology.

[12] Littman T, Kraus N, Mcgee T, Nicol T (1994). Binaural response patterns in subdivisions of the medial geniculate body. Brain research.

[13] Liégois-Chauvel C, Musolino A, Badier JM, Marquis P, Chauvel P. (1994). Evoked potentials recorded from the auditory cortex in man: evaluation and topography of the middle latency components. Electroencephalogr Clin Neurophysiol.

[14] Kraus N, Mcgee T, Karmos G, Molnär M, Csepe V, Czigler I, Desmedt JE (1995). Perspectives of Event-related Potentials Research.

[15] Mcgee T, Kraus N (1996). Auditory development reflected by middle latency response. Ear and Hearing.

[16] Kraus N, Kileny P, Mcgee T, Katz J (1999). Middle Latency Auditory Potentials.

[17] Arnfred SM, Eder DN, Hemmingsen RP, Glenthoj BY, Chen AC (2001). Gatting of the vertex somatosensory and auditory evoked potential P50 and the correlation to skin conductance orienting response in healthy men. Psychiatry Res.

[18] Ambrosini A, De Pasqua V, Afra J, Sandor PS, Schoenen J (2001). Reduced gating of middle-latency auditory evoked potentials (P50) in migraine patients:another indication of abnormal sensory processing?. Neurosci Lett.

[19] Miyamoto T, Miyamoto M, Takekkawa H, Kubo J, Hirata K, Katayama S (2001). A comparison of middle latency auditory-evoked response in obstructive sleep apnea syndrome before and after treatment. Psichiatry Clin Neurosci.

[20] Gerken GM, Hesse PS, Wiorkowski JJ (2001). Auditory evoked responses in control subjects and in patients with problem-tinnitus. Hear Res.

[21] Yvert B, Crouzeix A, Bertrand O, Seither-Preisler A, Pantev C (2001). Multiple supratemporal sources of magnetic and electric auditory evoked middle latency components in humans. Cereb Córtex.

[22] Yvert B, Fischer C, Guenot M, Krolak-Salmon P, Isnard J, Pernier J (2002). Simultaneous intracerebral EEG recordings of early auditory thalamic and cortical activity in human. Eur J Neurosci.

[23] Schochat E (2003). Resposta de latência média em crianças e adolescentes normo-ouvintes. Pró-Fono Revista de Atualização Científica.

[24] Arakawa K, Tomi H, Tobimatsu S, Kira J (2003). Middle latency auditory-evoked potentials in myotonic dystrophy: relation to the size of the CTG trinucleotide repeat and intelligent quotient. J Neurol Sci.

[25] Edmonds HL, Zhang YP, Shields CB (2003). New neurophysiology and central nervous system disfunction. Curr Opin Crit Care.

[26] Chaudhari L, Tandon OP, Vaney N, Agarwal N (2003). Auditory evoked responses in gestational diabetecs. Indian Physiol Pharmacol.

[27] Logi F, Fischer C, Murri L, Maughiere F (2003). The prognostic value of evoked responses from primary somatosensory and auditory córtex in comatose patients. Clin Neurophysiol.

[28] Boutros N, Korziuko O, Oliwa G, Feingold A, Campbell D (2004). Morphological and latency abnormalities of the mid-latency auditory evoked responses in schizophrenia: a preliminary report. Schzophr Res.

[29] Ghisolfi ES, Margis R, Becker J, Zanardo AP, Strimitzer IM, Lara DR (2004). Impaired P50 sensory gating in post-traumatic stress disorders secondary to urban violence. Int J Psycophyol.

[30] Pialarissi PR, Almeida MAO, Paiva Junior LEF, Silva A, Almeida SF (2006). Respostas auditivas evocadas de latência média: um estudo de padronização. Rev Bras Otorrinolaringol.

